# Prevalence, Risk Factors and Genotypes of *Giardia duodenalis* in Sheltered Dogs in Tuscany (Central Italy)

**DOI:** 10.3390/pathogens11010012

**Published:** 2021-12-23

**Authors:** Andrea Agresti, Federica Berrilli, Michela Maestrini, Isabel Guadano Procesi, Enrico Loretti, Niccolò Vonci, Stefania Perrucci

**Affiliations:** 1 Department of Veterinary Sciences, University of Pisa, 56124 Pisa, Italy; a.agresti@studenti.unipi.it (A.A.); michela.maestrini@phd.unipi.it (M.M.); 2 Department of Clinical Sciences and Translational Medicine, University of Rome “Tor Vergata”, 00133 Rome, Italy; berrilli@uniroma2.it (F.B.); Isabel.Guadano.Procesi@uniroma2.it (I.G.P.); 3 Department of Biology, Ph.D. Program in Evolutionary Biology and Ecology, University of Rome “Tor Vergata”, 00133 Rome, Italy; 4 Unità Funzionale Aziendale Igiene Urbana, Az. USL Centro Toscana, 50127 Firenze, Italy; enrico.loretti@uslcentro.toscana.it; 5 Unità Funzionale Igiene Epidemiologia e Sanità Pubblica, Azienda USL Toscana Centro, 50032 Borgo San Lorenzo, Italy; niccolo.vonci@uslcentro.toscana.it

**Keywords:** *Giardia duodenalis*, intestinal parasites, kennels, prevalence, genotypes, risk factors, central Italy

## Abstract

In sheltered dogs, the prevalence of *Giardia duodenalis* is frequently high and may include potential zoonotic genotypes. The prevalence, genotypes and potential risk factors of *G. duodenalis* were assessed in 168 dogs from four kennels (Pistoia, Prato, Florence, Valdarno) in Tuscany, central Italy and compared with data from previous Italian studies. The prevalence of other intestinal parasites was also investigated. Individual dog faecal samples collected from each kennel were examined by parasitological techniques and a rapid immunoassay for the detection of *G. duodenalis* and *Cryptosporidium* faecal antigens. On *Giardia*-positive samples, molecular analysis was performed for genotype identification. Overall, 69 dogs scored positive for *G. duodenalis* (41%), but significant differences (*p* ≤ 0.05) were found among the four kennels and sampling seasons. The potentially zoonotic assemblages A and B and the canine-specific assemblage C (Pistoia: A-AII, B, C; Prato: A-AII, B; Florence: A-AII; Valdarno: A and C) were identified. *Toxocara canis* (8.9%), *Trichuris vulpis* (3.6%), hookworms (1.19%) and *Cryptosporidium* sp. (0.6%) were also identified. The high prevalence of *G. duodenalis* and the identification of potentially zoonotic genotypes in all examined kennels underline the need to improve routine parasite monitoring and control measures and to provide insights into the zoonotic potential of *G. duodenalis*.

## 1. Introduction

*Giardia duodenalis* (syn. *Giardia intestinalis*, *Giardia lamblia*) is a worldwide-distributed intestinal flagellated protozoan that can infect domestic and wild animals, including dogs and humans [1,2]. The life cycle of *G. duodenalis* is direct and involves only two stages, the trophozoite and the cyst. Hosts may become infected via ingestion of infectious *G. duodenalis* cysts in contaminated food or water sources, or directly (cysts and trophozoites) via the faecal–oral route [1].

The localisation site of *G. duodenalis* is the small intestine, mainly the duodenum and jejunum, and the infection may be asymptomatic or may cause intestinal disease of different severity [3,4].

Molecular studies have shown that *G. duodenalis* includes eight distinct genotypes, also called assemblages, identified with the alphabetic letters from A to H [1]. Assemblages C and D are canine specific. Assemblages A and B are most frequently found in humans, but they can also infect other animals and are considered potentially zoonotic [5,6,7,8]. Assemblages A and B have been further divided into sub-assemblages (A-I to A-III and B-III, B-IV), some of which are more common in humans and others in animals [2,5]. Sub-assemblage A-I has been reported in humans and animals, while A-II is considered human specific but has also been identified in dog faecal samples [9,10]. Assemblage A-III has been detected exclusively in animals [5,11]. Although no definitive transmission between animals and humans has been documented, data from cross-sectional surveillance studies and assessments during giardiasis epidemics greatly support this possibility [9,12,13,14,15].

The prevalence of *G. duodenalis* infection in dogs varies depending on the population and area under examination, the diagnostic method used and the health status of the animals examined [16,17]. Young dogs, mainly puppies under 6 months of age, and some dog populations, such as stray or community-living animals, and breeding and sheltered dogs, show a higher risk of infection [4,8,16]. Furthermore, in conditions of high animal density and in highly contaminated environments, such as in shelters, repeated infections can be observed due to poor protection conferred by the immune system, continuous exposure to the parasite and continuous introduction of new animals [5,16,18].

In previous European studies [6,7,19,20], including most studies performed in Italy [21,22,23,24,25,26], the prevalence of *G. duodenalis* ranged between 20% and 57% in dogs.

In kennel dogs, canine host-specific assemblages C and D have been more frequently reported in European countries, such as the UK and Spain, but these animals can also be infected by the zoonotic assemblages A and B [7,19]. These data agree with those from sheltered dogs in Italy [21,22,23,24,25], in which assemblages C and D were found to be prevalent, but assemblage A was also observed. However, assemblage B was identified only in a single kennelled dog in northern Italy [26].

Feng and Xiao [2] found that an increase in *G. duodenalis* zoonotic assemblages is observed in dogs when human–animal contacts are high. In fact, it has been highlighted that two transmission cycles may be present in urban environments with the transmission of canine-specific assemblages between dogs and potential transmission of zoonotic assemblages, mainly assemblage A, between dogs and humans [1,2,5,13,27,28]. The transmission of dog-specific genotypes is thought to be favoured by intense contact among dogs living in large communities, competing with the transmission of other genotypes, especially in crowded kennels with poor hygiene [1,2,5]. However, zoonotic *G. duodenalis* assemblages (A and B) and sub-assemblages (AII, BIII and BIV) have been increasingly documented among kennel dogs in Europe in recent years [3,6,19,25].

The objectives of this study were to evaluate the prevalence and potential contributing factors, such as age, shelter of provenance and seasonality, of *G. duodenalis* in kennel dogs in Tuscany (central Italy); to identify the genotypes of *G. duodenalis* isolates; and to compare obtained data with those reported in dogs from Italy.

## 2. Results

### 2.1. Parasitological and Immunological Analysis

Overall, 69 out of 168 examined dogs scored positive for *G. duodenalis*, with a prevalence of 41.07%. The highest prevalence of *G. duodenalis* was found among puppies younger than 6 months of age (8/11 dogs, prevalence 72.73%), followed by the 6–12 month (18/28 dogs, prevalence 64.29%), 1–8 year (27/78 dogs, prevalence 34.62%) and >8 year (16/51 dogs, prevalence 31.37%) age groups. However, these differences were found to be significant (*p* < 0.05) only at univariate statistical analysis (Table 1).

Among the examined kennels, in the kennel in Pistoia (25/33 dogs, prevalence 75.76%) the prevalence of *G. duodenalis* was significantly higher (*p* < 0.05) compared with that found in other shelters, while the lowest prevalence was found in the Florence kennel (13.64%, 3/22 dogs) (Table 1). The prevalence of *G. duodenalis* found in the kennels of Valdarno and Prato was 33.85% (22/65 dogs) and 39.58% (19/48 dogs), respectively.

The number of dogs positive for *G. duodenalis* was high in all seasons, namely 27/50 dogs in autumn (54%), 16/50 dogs in winter (32%), 19/52 dogs in spring (36.54%) and 7/9 dogs (77.78%) in summer. However, using multivariate analysis, the prevalence in winter was observed to be significantly lower (*p* < 0.05) than in other seasons (Table 1).

Concerning the other identified intestinal parasites, a total of 23 dogs were found positive for intestinal nematodes (23/168 dogs, 13.69%). More specifically, *Toxocara canis* was identified in 15 dogs (15/168, prevalence 13.69%), *Trichuris vulpis* in 6 dogs (6/168, 3.57%) and hookworms (*Ancylostoma caninum*/*Uncinaria stenocephala*) in 2 dogs (2/168, 1.19%). *T. canis* was present in all examined kennels. A single dog (>8 years) from the Florence kennel was found positive for *Cryptosporidium* sp. (1/168, 0.6%).

Multiple parasite infections were detected in 14 dogs found to be infected by 2 or 3 parasite species (14/168, 8.33%). Specifically, 13 dogs were found to be infected by *G. duodenalis* and *T. canis*/hookworms/*T. vulpis* (13/168, 73.74%), while a single dog scored positive for *G. duodenalis*, *Cryptosporidium* sp. and *T. canis* (1/168, 0.6%).

### 2.2. Molecular Analysis

Molecular analysis was performed on 25 out of the 69 *Giardia*-positive isolates, and positivity was confirmed in all of them. Sequence analysis of the *SSU-rRNA* gene fragment allowed assignment of the isolates to the potentially zoonotic assemblages A and B and to the dog-specific assemblage C (Table 2). A total of 16 isolates were assigned to assemblage A, 5 of which were identified as sub-assemblage AII (3 at *β-giardin* locus and 2 at *tpi* locus); 6 to assemblage B; and 2 to assemblage C. Based on the double peaks at the diagnostic positions, a single sample was found concurrently positive for assemblages A and assemblage B (Table 2). *G. duodenalis* genotypes identified in each kennel were A-AII, B and C in the shelter of Pistoia; A-AII and B in the shelter of Prato; A-AII in the shelter of Florence; and A and C in the shelter of Valdarno.

Table 2 shows data regarding *G. duodenalis* genotypes identified in this study and previously reported in dogs in different areas of Italy.

## 3. Discussion

The prevalence of canine *G. duodenalis* infection is highly variable, greatly dependent on the immune status, age and lifestyle of animals, and on the geographical area examined [17]. The mean prevalence of *G. duodenalis* in dogs appears to be about 15% [17], but it can be higher than 45% [11]. Higher prevalence rates in kennel or shelter dogs compared to household or owned dogs are widely evidenced worldwide [36,37], with prevalence ranging from 20% to 59% in Europe [6,7,19,20], as in almost all the studies performed in Italy [21,22,23,24,25,26,38]. The overall prevalence of *G. duodenalis* found in the dogs included in this study (41.07%) was extremely high, and it was high among all kennels examined, therefore confirming the high prevalence previously reported in shelter dogs in the European territory.

It has been observed in many studies that dogs younger than one year of age are more likely to be positive for *G. duodenalis* [16,17,22,30,32,38,39]. This observation was confirmed in this study, which highlighted a significantly higher prevalence in puppies under 6 months of age (72.73%) and in subjects of the 6–12 month age group (64.29%) compared to older age groups. However, the statistical significance of this factor emerged only at the univariate analysis due to the small number of young subjects examined in this study.

Concerning the season, multivariate logistic regression analysis evidenced that the risk of *G. duodenalis* infection was lower in winter than in other seasons. This result disagrees with those found in some previous studies carried out in the same region (Tuscany) and in other geographical areas, in which both privately owned and sheltered dogs were considered [40,41,42]. Seasonal differences in the management of the kennels here examined, a higher parasite spread in some seasons compared to other seasons and, lastly, seasonal environmental conditions that may favour the possibility for infected dogs to have more contact with other animals may represent some potential factors explaining this result.

Among the examined shelters, the prevalence found in the Pistoia kennel in this study was significantly higher (about 75.8%) compared to that observed in the other kennels previously examined in the European territory. Moreover, the prevalence of *G. duodenalis* in the Pistoia kennel was characterised by high rates in all seasons of the year and in all animal age groups. These findings may suggest that a number of factors may contribute to the high prevalence of *G. duodenalis* in this kennel. Overcrowding is considered a favourable factor for the spread of this protozoan parasite [3,8,16]. Effective sanitisation and control measures may also be more difficult to perform in these conditions. Therefore, a reduction in animal density and improvement in hygienic and control measures are advised in this kennel. Nevertheless, hygiene and effective control measures should be improved in all examined kennels, as in all of them, *G. duodenalis* prevalence was high. Moreover, in this study, other parasites, including potentially zoonotic species, such as *T. canis*, *Cryptosporidium* sp. and hookworms [24,43], were often found to be associated with *G. duodenalis*.

Among effective measures, diagnostic procedures and treatment of new entry animals, periodic parasite monitoring, cleaning, disinfection of the premises and dog walking areas with effective antiparasitic products, and washing of dogs after treatment for *G. duodenalis* have been recommended [16,44].

The confirmation of the molecular positivity for *G. duodenalis* and the high prevalence of potentially zoonotic assemblages identified in shelters here examined underline the importance of this parasite as an additional potential public health risk. In fact, the zoonotic assemblage A was identified in all examined kennels and the human-specific sub-assemblage AII in three of them. The zoonotic assemblage B was also present in two kennels, but sequences from assemblage B isolates were characterised by larger genetic variability and by the presence of heterogeneous positions, which made assignment to specific genotypes difficult, as evidenced in other studies [45]. Assemblage C was identified only in two kennels. Furthermore, multiple genotypes (2 or 3) were found concurrently present in each kennel.

In previous studies carried out in Italy in different dog populations, the canine-specific assemblages C and D were found to be highly prevalent [25,26,30,46]. However, the potentially zoonotic assemblage A was additionally identified [23,25,30], while the zoonotic assemblage B has been rarely detected in dogs in Italy [26]. Therefore, the high prevalence of potentially zoonotic genotypes and the frequent finding of assemblage B in this study do not agree with previous data on dogs in Italy. However, assemblage B was found to be prevalent in sheltered dogs in a recent study in Spain [19].

From the detailed examination of the genotypes identified in the different dog populations examined in the various studies performed in Italy, including the present study, a high variability in the frequency of canine and potentially zoonotic genotypes can be observed. More specifically, it emerges that in some studies, potentially zoonotic genotypes were [29,30] or were not [24,32,34,35] identified among privately owned dogs, while in other studies, potentially zoonotic genotypes appeared to be prevalent or showed a high prevalence among kennel or stray dogs [23,25], or were almost completely absent [26,33]. Moreover, this variability additionally emerged by the comparison of *G. duodenalis* genotypes in the different kennels here examined, as potentially zoonotic genotypes were identified in all of them, while canine genotypes were present in only two of them.

The reasons for the high variability of *G. duodenalis* genotypes found in kennels in previous studies and in this study may depend on numerous factors related to kennel management, genotypes infecting new entry dogs and the possibility that these genotypes may spread to other dogs.

Therefore, the findings from this study confirm data from other recent studies [3,6,25] showing that the transmission of potentially zoonotic genotypes is not limited to household dogs but can also be high in dogs living in large communities, as in sheltered dogs, in which the presence of dog-specific genotypes was thought to be favoured and competing with the transmission of other genotypes [1,2,5,28]. The increased occurrence of zoonotic assemblages in dogs in recent years has been suggested to be the consequence of more frequent cross-species transmission of *G. duodenalis* between humans and dogs [5].

## 4. Materials and Methods

### 4.1. Animals and Kennels

From September 2018 to July 2019, a total of 168 dogs of different ages (2 months–17 years) were included in the study. Dogs lived in four different shelters from the Northern region of Tuscany (central Italy), namely, the kennels of Valdarno (65 dogs), Pistoia (48 dogs), Prato (33 dogs) and Florence (22 dogs).

For the control of parasites, in the examined kennels, all dogs are treated at the time of their entry and about once a year with a commercial anthelmintic drug containing febantel, pyrantel and praziquantel.

In all these shelters, dogs are single caged. Cleaning and disinfection are performed by using chlorine-based disinfectant solutions.

### 4.2. Sampling

Individual faecal samples were collected from all examined dogs. In all kennels, sampling was performed four times/year to include the four seasons: autumn (October–December 2018), winter (January–March 2019), spring (April–May 2019) and summer (June–July 2019). Each dog was sampled only once.

Collection of samples and manipulation of animals were authorised by the shelters and the Italian Ministry of Health in the framework of the Italian surveillance programs for potential zoonotic diseases (Italian law No. 281-1991).

### 4.3. Parasitological and Immunological Analysis

Faecal samples were examined using a commercial rapid immunoassay to detect *Giardia* spp. and *Cryptosporidium* spp. faecal antigens (RIDASCREEN^®^ *Cryptosporidium*/*Giardia* Combi, R-Biopharm, Darmstadt, Germany). For the identification of helminthic eggs and cysts/oocysts of protozoa, all faecal samples were analysed microscopically by the Mini-FLOTAC technique [47].

### 4.4. Molecular Analysis

On a sub-group of *Giardia*-positive samples (25/69, 3-8/shelter), which included those samples from each kennel found strongly positive during the immunoassay, molecular analysis was performed to identify the species and genotypes of *Giardia*. For DNA extraction, samples were processed using a commercial kit (QIAamp DNA Stool Mini Kit, QIAGEN, Valencia, CA, USA). PCR protocols were applied to amplify fragments of the small subunit ribosomal RNA (*SSU rRNA*, 130 bp) [48], of *β-giardin* (*β-giardin*, 384 bp) [49] and of the triose phosphate isomerase (*tpi*, 530 bp) [50] genes. Positive amplicons were purified using mi-PCR Purification Kit, Metabion International AG. Amplification products were sent to an external laboratory for sequencing (Bio-Fab Research, Rome, Italy). Forward and reverse sequences were manually checked using Finch TV 1.4 software (Geospiza, Inc., Seattle, WA, USA). The obtained consensus sequences were then compared with those available on GenBank database using the Standard Nucleotide BLAST search.

### 4.5. Statistical Analysis

Statistical analysis was performed with Stata^®^ v12.0 (Stata Corp., College Station, TX, USA) and the significance level was set at *p* < 0.05.

The chi square test was used to assess any statistical differences in the prevalence of *G. duodenalis* according to the age and kennel of provenance of dogs and sampling season. According to age, dogs were divided into four groups: ≤6 months, 6–12 months, 1–8 years and >8 years.

Multivariate logistic regression analysis was used to evaluate potential statistical correlations between *G. duodenalis* and the selected independent variables (age, kennel of provenance and season).

## 5. Conclusions

The high prevalence of *G. duodenalis* and the identification of potentially zoonotic genotypes in all examined kennels underline the need to monitor sheltered dogs for this infection, to improve routine *Giardia* control measures in kennels and to provide insights into the zoonotic potential of *G. duodenalis* in sheltered dogs.

## Figures and Tables

**Table 1:** Results of multivariate logistic regression analysis regarding the prevalence of *Giardia duodenalis* in different kennels, seasons and age groups.

	Odds Ratio	*p*	Confidence Interval 95%
**Kennels**			
Pistoia	1		
Firenze	0.057	0.000 *	0.012–0.266
Prato	0.215	0.004 *	0.075–0.620
Valdarno	0.167	0.000 *	0.006–0.456
**Age**			
0–6 months	1		
6–12 months	0.716	0.702	0.129–3.965
1–8 years	0.267	0.098	0.056–1.273
>8 years	0.246	0.086	0.050–1.222
**Season**			
Autumn	1		
Winter	0.338	0.021 *	0.134–0.851
Spring	0.511	0.137	0.211–1.237
Summer	0.785	0.718	0.211–2.921

* Statistically significant values; the significance level was set at *p* < 0.05.

**Table 2:** Genotypes of *Giardia duodenalis* in dogs from Italy in previous studies and in the present study.

Region	Dog Population	N. Dogs	Assemblage (%)	Locus	Time Tested (Year)	References
Lombardy region (Northwestern Italy) and Veneto region (North-eastern Italy)	Kennel and privately owned dogs	21	A (28.6%) C (4.7%) D (61.9%) A + D (4.7%)	*β-giardin*	2003	Lalle et al., 2005 [29]
Abruzzo region (Central Italy)	Kennel and privately owned dogs	240	C (10%, only in privately owned dogs) D-D1 (83.3%) A-AI (6.7% only in privately owned dogs)	*SSU-rRNA + β-giardin*	2003–2005	Paoletti et al., 2008 [22]
Latium (Central Italy)	Kennel dogs	127	A (30.7) C (53.8) D (15.3)	*SSU-rRNA*	2005–2006	Scaramozzino et al., 2009 [23]
Apulia (Southern Italy)	Free-roaming dogs in a Rom camp	14	AI (100%)	*β-giardin*	unspecified	Marangi et al., 2010 [13]
Tuscany (Central Italy)	Privately owned dogs	239	C (77.8%) A (22.2%)	*SSU-rRNA + gdh*	2008–2010	Riggio et al., 2013 [30]
Sardinia (Southern Italy)	Privately owned dogs and kennel dogs	655	C (36.1%) D (49%) AII (4.2%)	*SSU-rRNA + β-giardin*	2007–2010	Pipia et al., 2014 [31]
Lombardy region (Northwestern Italy)	Privately owned dogs	253	C (54.5%) D (45.5%).	*SSU-rRNA*	2010–2011	Zanzani et al., 2014 [32]
Veneto and Friuli-Venezia Giulia regions (Northeastern Italy)	Shelter dogs	318	C (46.23%, 49/106) D (27.36, 29/106) B1 (0.94%, 1/106)	*SSU-rRNA*	2008–2012	Simonato et al., 2015 [26]
Central Italy	Privately owned and kennel dogs	502	C (75%, 15/20) D (10%, 2/20) C+D (15%, 3/20)	*SSU-rRNA*	2011–2013	Paoletti et al., 2015 [24]
Latium region (Central Italy)	Stray dogs	262	C (24%) D (76%)	*SSU-rRNA*	2014–2015	De Liberato et al., 2018 [33]
Tuscany and Latium regions (Central Italy)	Shelter dogs	639	A (33%) C (67%)	*SSU-rRNA*	2011–2014	Sauda et al., 2018 [25]
Tuscany region (Central Italy)	Privately owned dogs	47	C (83.3%, 5/6) D (16.7%, 1/6)	*SSU-rRNA*	2016–2017	Perrucci et al., 2020 [34]
Campania region (Southern Italy)	Privately owned dogs	24	D (100%, 6/6)	*β-giardin*	2018–2019	Ciuca et al., 2021 [35]
Tuscany region (Central Italy)	Shelter dogs	168	C (8%, 2/25) A [64%, 16/25 (of which 20%, 5/25 AII)] B (24%, 6/25) A+B (4%, 1/25)	*SSU-rRNA +* *β* *-giardin + tpi*	2018–2019	Present study

## Data Availability

Not applicable.
